# Dual anti-thrombotic treatment with direct anticoagulants improves clinical outcomes in patients with Atrial Fibrillation with ACS or undergoing PCI. A systematic review and meta-analysis

**DOI:** 10.1371/journal.pone.0235511

**Published:** 2020-07-09

**Authors:** Salvatore De Rosa, Jolanda Sabatino, Alberto Polimeni, Sabato Sorrentino, Ciro Indolfi

**Affiliations:** 1 Division of Cardiology, Department of Medical and Surgical Sciences, "Magna Graecia" University, Catanzaro, Italy; 2 Cardiovascular Research Center, "Magna Graecia" University, Catanzaro, Italy; Universita degli Studi di Napoli Federico II, ITALY

## Abstract

**Background:**

There is recent new evidence regarding the combined use of direct oral antiocoagulants and antiplatelet agents in patients with Atrial Fibrillation undergoing PCI.

**Purpose:**

To compare the efficacy of dual antithrombotic treatment (DAT) including a direct oral anticoagulant (DOAC) and an antiplatelet agent versus triple antithrombotic treatment (TAT) with a vitamin K antagonist (VKA).

**Data sources:**

PubMed, SCOPUS and Google Scholar from through 09/09/2019; references of eligible studies; relevant scientific sessions abstracts and cardiology websites.

**Study selection:**

Randomized controlled trials that compared DAT including a DOAC with TAT including a VKA and that reported at least the rates of stroke, Stent thrombosis and bleeding.

**Data extraction:**

Two investigators independently extracted study data and assessed study quality.

**Data synthesis:**

Four randomized trials that compared DAT including a DOAC with TAT including a VKA were available. Among these, one trial included two independent treatment arms with different DOAC dose, both compared against TAT. For this reason, the two arms were treated independently, resulting in 5 randomized comparisons available for meta-analysis, with a total of 8654 patients involved. The primary safety endpoint was significantly lower in the DAT arm (14.4%) compared to the TAT arm (23%) (RD = -0.08; p<0.001). In addition, we found no significant difference in the incidence of stroke between the treatment arms (p = 0.23). Similarly, no significant difference in the incidence of Stent Thrombosis between the treatment arms (p = 0.08).

**Limitations:**

All trials included were open-label, even though data were blindly analyzed. Qualifying criteria are heterogeneous.

**Conclusions:**

Compared TAT including a VKA, a therapeutic DAT regimen including a DOAC was associated with a significant reduction of the primary safety endpoint in AF patients undergoing PCI with stent implantation for an ACS or chronic coronary syndrome, while no significant difference was found in the rate of ischemic adverse events, including stroke, acute myocardial infarction or stent thrombosis.

## Introduction

Current management of patients with Atrial Fibrillation experiencing an Acute Cardiovascular Syndrome or undergoing non-delayable percutaneous coronary interventions is challenging. In fact, the combination of dual antiplatelet treatment with oral anticoagulation is associated to a substantial increase of bleeding complications [1].

Dual antithrombotic treatment including a direct oral anticoagulant (DOAC) and single antiplatelet treatment with a P2Y12 inhibitor was associated with a significant increase in safety, as both total and major bleeding were significantly reduced using this approach in the recent PIONEER-AF Trial and RE-DUAL PCI Trial [2–3]. Nevertheless, concerns remained about the efficacy of this dual treatment approach to prevent thromboembolic or ischemic events. In fact, both studies were underpowered to test the efficacy of the dual drug approach versus the triple drug approach for the prevention of stroke or systemic embolism. Concerns around this issue have further intensified with the finding of a trend towards an increased rate of stent thrombosis in the dual treatment arm of the RE-DUAL PCI trial [4].

More recently, the results of the AUGUSTUS and ENTRUST AF-PCI trials provided additional evidence [5–6]. The AUGUSTUS and ENTRUST AF-PCI had similar findings compared to the previous PIONEER-AF PCI and RE-DUAL PCI studies. Yet, despite being underpowered to test the efficacy for prevention of stroke or systemic embolism as the two previous trials, the 8654 patients enrolled to date in the four RCT available [2–3,5–6] allow a powered comparison for the efficacy endpoint. Hence, we designed a meta-analysis to assess both safety and efficacy of a dual antithrombotic regimen to the triple antithrombotic treatment in patients with AF experiencing an ACS or undergoing non-delayable PCI.

## Methods

We developed a protocol for the review June 26^th^ 2019 and registered it in PROSPERO (http://www.crd.york.ac.uk/PROSPERO/display_record.php?ID=CRD149984) on September 9^th^ 2019.

### Data sources and searches

We searched PubMed, SCOPUS and Google Scholar electronic databases from December 1^st^ 2004 through September 9^th^ 2019 using the following keywords and the corresponding MeSH terms: “PCI”, “ACS”, “atrial fibrillation”, “DOAC/NOAC”. We also checked the reference lists of eligible studies and screened scientific abstracts and relevant websites (www.clinicaltrialresults.org, www.escardio.org, www.tctmd.com, https://accscientificsession.acc.org/, https://exhibitatsessions.org/).

### Study selection

Two investigators (JS, SDR) independently screened search records to identify eligible trials. There were no disagreements. Randomized controlled trials were included if comparing a DAT including a DOAC versus a TAT with VKA. Additional inclusion criteria were to report at least the following outcomes: major bleeding, stroke and stent thrombosis (ST). Exclusion criteria were duplicate publications, and trials published in a language other than English or where the pre-specified endpoint measure was not reported.

### Data extraction and quality assessment

Two reviewers (JS, SDR) independently extracted data about study characteristics and event rates from full articles. Two investigators (SDR, JS) independently assessed study quality using the Cochrane Risk of Bias Tool (http://methods.cochrane.org/bias/assessing-risk-bias-included-studies). In particular, randomization method, allocation concealment, blinding of patient, investigator, and outcome adjudication committee, reporting bias, attrition bias, and any other potential source of bias, such as those related to trial designs, or the risk for contamination or cross-over between the groups were assessed.

### Data synthesis and analysis

We data extracted from the original primary publications [2–3,5–6]. We based our primary analyses on the composite safety endpoint of major bleeding events, while the primary efficacy endpoint was a composite of major adverse cardiovascular events. In addition, stroke, stent thrombosis and death were analyzed as secondary endpoints. We used the Risk Difference (RD) with 95% confidence interval as the summary measure [7]. The random-effects model was used to compute estimates for the summary effect [8]. Heterogeneity was assessed using the Cochrane Q test by means of a chi-squared function. P values below 0.10 were considered indicative for heterogeneity. I^2^ values were calculated for estimation of variation among studies attributable to heterogeneity. Meta-analysis results were displayed with Forest plots where the measure of effect (RD) for each study is represented by a square and the area of each square is proportional to study weight.

A meta-regression analysis was performed to examine a potential association between time from index procedure to randomization, time at therapeutic target and proportion of ACS patients on bleeding complications. Subgroup and sensitivity analyses were conducted using fixed effect and random effects models alternatively [9–11]. Analyses were performed using Review Manager 5.4 and OpenMetaAnalyst 10. Statistical power was calculated using the previously described method for random effects meta-analyses [12].

## Results

**Fig 1:**
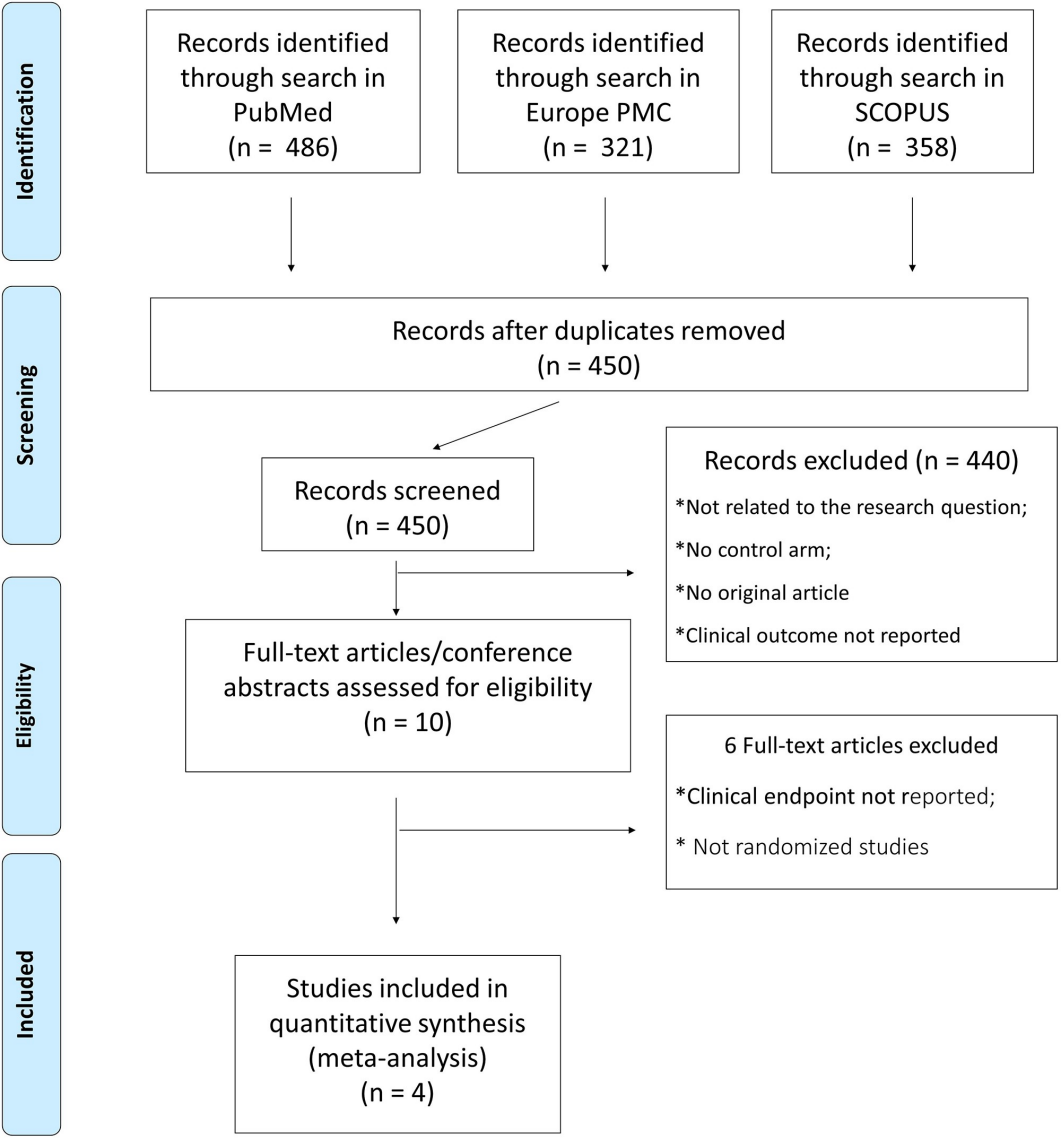
Study selection flowchart.

Of 450 screened records, we identified 4 individual trials that compared a combined dual therapy including ASA and a Direct Oral Anticoagulant (DOAC) versus a triple therapy including two antiplatelet agents and warfarin (Fig 1) [2–3,5–6]. All trials were multi-centre open label studies. All trials were funded by industry. The PIONEER AF-PCI trial (Open-Label, Randomized, Controlled, Multicenter Study Exploring Two Treatment Strategies of Rivaroxaban and a Dose-Adjusted Oral Vitamin K Antagonist Treatment Strategy in Subjects with Atrial Fibrillation who Undergo Percutaneous Coronary Intervention) randomized patients within 72h after PCI to one of the following arms in a 1:1:1 ratio: i) low-dose rivaroxaban (15 mg once daily) plus a P2Y12 inhibitor for 12 months (group 1), ii) very-low-dose rivaroxaban (2.5 mg twice daily) plus DAPT for 1, 6, or 12 months (group 2), iii) standard therapy with a vitamin K antagonist plus DAPT for 1, 6, or 12 months (group 3) [2]. One of the two TAT arm of PIONEER AF-PCI (ASA + P2Y12 inhibitor + rivaroxaban 2.5mg twice daily) was excluded from the analysis as the rivaroxaban 2.5mg dose is not approved for prevention of thrombo-embolism in AF patients [2]. In the RE-DUAL, an open-label, multicentre randomized trial, dabigatran was compared to TAT with VKA in 2,725 patients. The RE-DUAL tested two different DOAC doses in parallel [3]. Hence, the two dose-arms were treated independently for calculation of meta-analyses. The AUGUSTUS trial tested the clinical impact of treatment with apixaban with and without aspirin in 4,614 patients with AF and an acute coronary syndrome or undergoing elective PCI [5]. The study was designed with a double randomization scheme of apixaban versus VKA and ASA versus placebo. The ENTRUST AF-PCI trial included 1506 patients with AF after successful PCI, 52% of whom had an ACS. Patients were randomly assigned to either edoxaban + P2Y12 inhibitor, or VKA with DAPT [6]. Edoxaban was given at dose approved for stroke prevention dose (60 mg once daily). Clopidogrel was the P2Y12 inhibitor of choice in 92% patients. The median duration of TAT was 66 days (IQR 33–188).

Characteristics of the trials are presented in Table 1.

**Table 1:** Randomized clinical trials assessing DAT including a DOAC versus TAT including VKA in patients with atrial fibrillation undergoing PCI.

Trial name	PIONEER AF-PCI	RE-DUAL PCI	AUGUSTUS	ENTRUST AF-PCI
**Trial type**	Randomized, open label	Randomized, open label	Randomized, open label	Randomized, open label
**Study enrollment**	May 2013-July 2015	July 2014-May 2017	Sep 2015-April 2018	Feb 2017-May 2018
**Major inclusion criteria**	Age ≥ 18 years with non-valvular AF within last 1 year and who had just undergone PCI with stent placement (bare metal or drug eluting) for stable angina or acute coronary syndrome	Age ≥ 18 years with non-valvular AF within last 1 year and who had just undergone PCI with stent placement for stable angina or acute coronary syndrome	Age ≥ 18 years with previous, persistent, permanent or paroxysmal non-valvular AF; recent acute coronary syndrome or PCI, planned use of P2Y12 inhibitor for at least 6 months	Age ≥ 18 years, atrial fibrillation requiring oral anticoagulation, successful PCI for stable CAD or ACS
**Major exclusion criteria**	History of stroke/TIA, significant gastrointestinal bleeding within 12 months before randomization, eGFR<30 ml/min, Hgb<10 g/dl	Mechanical or biological heart valves, cardiogenic shock, prior stroke, surgery, gastrointestinal bleeding, major bleeding within 1 month prior to randomization, Hgb<10 g/dl, eGFR<30 ml/min, active liver disease	History of intracranial haemorrhage, recent or planned CABG, coagulopathy, ongoing bleeding, contraindication to either VKA, apixaban, P2Y12 inhibitors or aspirin; severe renal insufficiency	Mechanical heart valves, moderate-to-severe mitral stenosis, end-stage renal disease, other major comorbidities
**Treatment arm**	Rivaroxaban + single antiplatelet therapy with clopidogrel/ prasugrel/ticagrelor for 12 months duration	Dabigatran 110 mg twice daily or 150 mg twice daily + clopidogrel/ticagrelor	Two-by-two factorial design: Apixaban vs VKA (target INR = 2.0–3.0); ASA 81 mg/die vs placebo	Edoxaban 60 mg once daily + clopidogrel 75 mg for 12 months by default: alternatively prasugrel 5/10 mg once daily or ticagrelor 90 mg twice daily at the investigator’s discretion
**Control arm**	ASA + clopidogrel/prasugrel/ ticagrelor + warfarin, duration 1, 6, or 12 months pre-specified per treating physician	Aspirin + clopidogrel/ ticagrelor + warfarin	See cell above for randomized treatments. In addition clopidogrel/prasugrel/ticagrelor for 6 months	VKA + clopidogrel 75 mg for 12 months by default: alternatively prasugrel 5/10 mg once daily or ticagrelor 90 mg twice daily at the investigator’s discretion + ASA 100 daily for 1–12 months
**Follow up, months (mean)**	12 months	14months	6 months	12 months
**Primary outcome (primary safety endpoint)**	Clinically significant bleeding (a composite of TIMI major and minor bleeding, or bleeding requiring medical attention)	ISTH major bleeding or clinically relevant non-major bleeding event	ISTH major or clinically relevant nonmajor bleeding	Composite of major or clinically relevant non-major (CRNM) bleeding (according ISTH)
**Secondary outcome (secondary efficacy endpoint)**	MACE (a composite of death, MI or stroke), each component of MACE and stent thrombosis	Composite of death, MI, stroke, systemic embolism or unplanned revascularization	A composite of death or hospitalization; a composite of death or ischemic events; each component of the composite endpoints; Acute Myocardial Infarction, Urgent revascularization and stent thrombosis	Stroke, ischemic stroke, haemorrhagic stroke, systemic embolic events, MI, all-cause death, cardiovascular or unexplained death

Risk of bias assessments are reported in S1 Table. All trials used an adequate method of randomization and allocation concealment. Blinding of patients and care givers was not possible, as no sham procedure was performed in the medical treatment group in any of the trials. However, endpoint adjudication committees were blinded to the treatment strategy in all trials. The risk for selection bias, detection bias, attrition bias and reporting bias was judged as low. The risk for performance bias was present.

### Measures of safety

**Fig 2:**
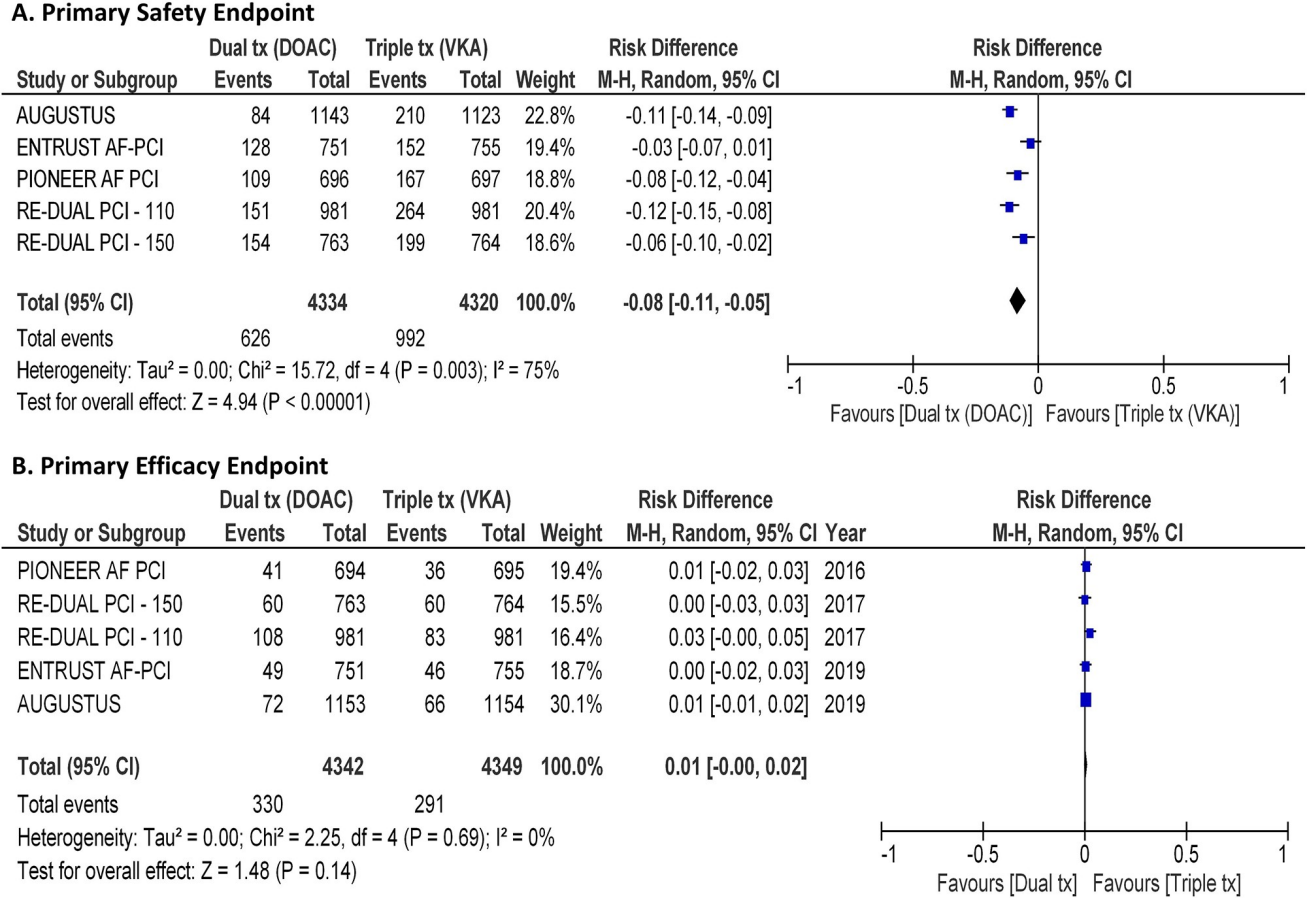
Primary endpoints. Forest plots illustrating the results of meta-analysis on the primary safety endpoint (**panel A**), and the primary efficacy point (**panel B**). 95% C.I. = 95% confidence interval; DOAC = direct oral anticoagulants; VKA = vitamin K antagonist; I^2^ = Inconsistency index.

The primary safety endpoint, a trial-defined composite endpoint of major bleeding events, was registered in 14.4% of patients treated with a DAT including a DOAC compared with 23% treated with TAT including VKA (RD, -0.08, 95% CI, -0.11 to -0.05; p<0.001; I^2^ = 75%; Fig 2A).

### Measures of efficacy

**Fig 3:**
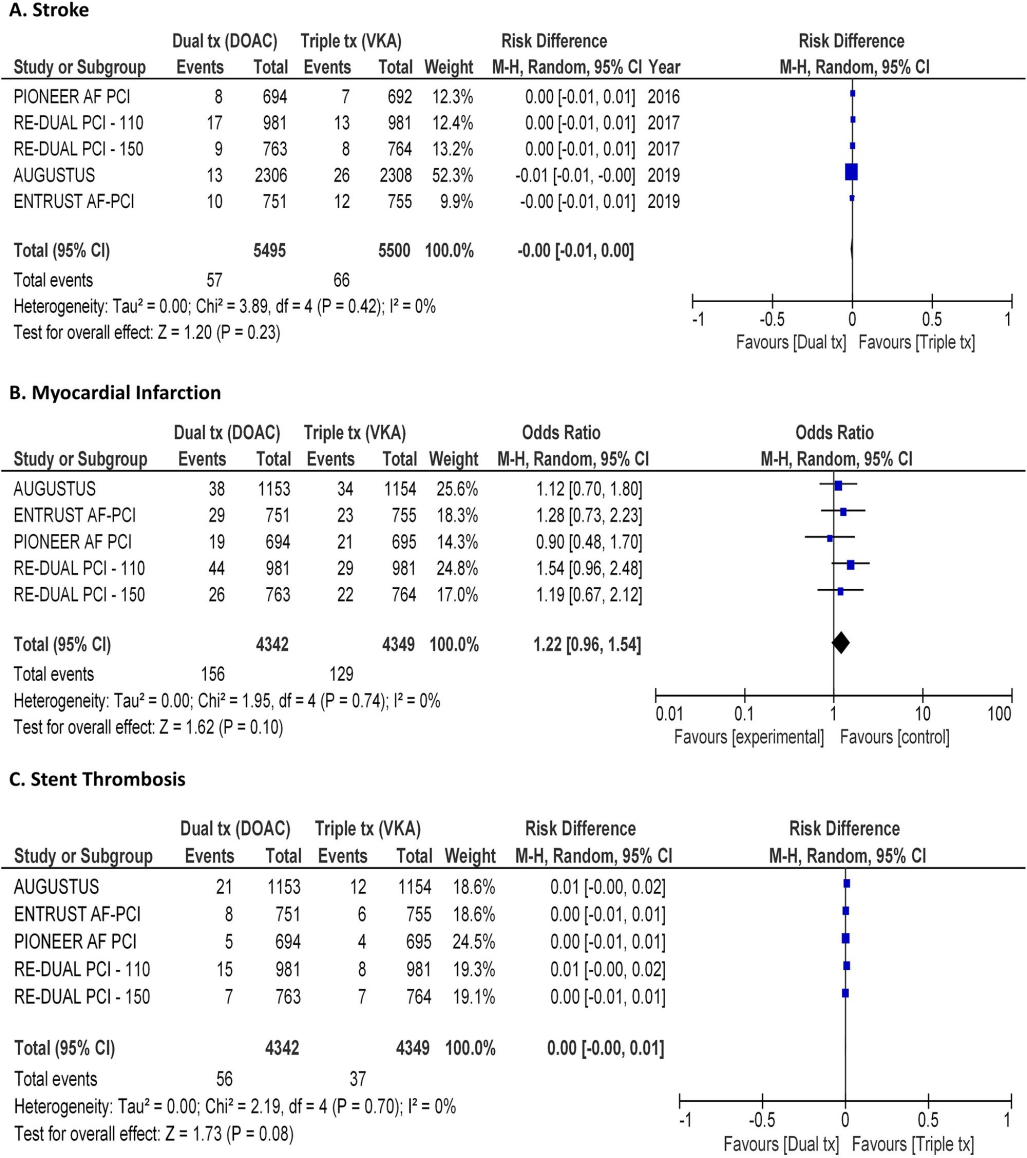
Forest plots illustrating the results of meta-analysis on the rate of stroke (panel A), myocardial infarction (panel B) and stent thrombosis (panel C). 95% C.I. = 95% confidence interval; DOAC = direct oral anticoagulants; VKA = vitamin K antagonist; I^2^ = Inconsistency index.

The primary efficacy endpoint, a trial-defined composite endpoint of major adverse cardiovascular events (MACE), was registered in 7.6% of patients treated with a DAT including a DOAC compared with 6.7% treated with TAT including VKA (RD, 0.01, 95% CI, -0.00 to 0.02; p = 0.14; I^2^ = 0%; Fig 2B). A stroke occurred in 1.4% of patients treated with a DAT including a DOAC compared with 1.3% treated with TAT including VKA (RD, -0.00, 95% CI, -0.01 to 0.00; p = 0.23; I^2^ = 0%; Fig 3A). Acute myocardial infarction was registered in 3.6% of patients treated with a DAT including a DOAC compared with 3% treated with TAT including VKA (RD, 0.01, 95% CI, -0.00 to 0.01; p = 0.13; I^2^ = 0%; Fig 3B). Stent thrombosis was registered in 1.3% of patients treated with a DAT including a DOAC compared with 0.9% treated with TAT including VKA (RD, 0.01, 95% CI, -0.00 to 0.01; p = 0.08; I^2^ = 0%; Fig 3C). Interestingly, power calculation yielded a power 0,90 for this latter comparison.

### Meta-regression and subgroup and sensitivity analyses

Heterogeneity was very low across all endpoint analysed except for bleeding, the primary endpoint of this analysis. At subgroup analysis it emerged that most heterogeneity was raising from the 3 studies using full-dose DOACs (RE-DUAL PCI 150 mg, AUGUSTUS, ENTRUST AF-PCI), with an I^2^ = 83% and a Q = 11.8 with 2 degrees of freedom (p = 0.003). No significant heterogeneity was found within the low-dose DOACs subgroup (Q = 1.3; p = 0.250). At meta-regression, a larger effect was found with increasing proportion of ACS patients included in single studies (p<0.001). An interaction was also present with the time from index procedure to randomization (p = 0.002), as well as with the time with INR below 2 (p = 0.003). Meta-analysis results were similar when compared using the fixed effect or random effects models.

## Discussion

Results of this meta-analysis demonstrate that a DAT regimen including a DOAC prevents bleeding events in patients with atrial fibrillation undergoing PCI, compared to a TAT regimen including a VKA. Our main results are: i) DAT significantly reduces by 40% bleeding complications compared to TAT (p<0.0001); ii) no significant difference is evident in the rate of Major Adverse Cardiovascular events (p = 0.14); iii) incidence of Stroke was comparable between the treatment arms and no statistically significant difference was found (p = 0.23); iv) no difference in the incidence of Stent Thrombosis was found between the two treatment strategies (p = 0.08).

These results provide an additional piece of information in this topic. In fact, summing up the results of the four randomized studies results in a higher statistical power to test this pressing clinical question, i.e. whether dual antithrombotic treatment including a direct oral anticoagulant can warrant adequate prevention of stroke or systemic embolism. In fact, the availability of nearly 10,000 patients, enrolled in the trials mentioned above allows a more reliable comparison against the triple treatment strategies, showing that using a Dual tx including it is possible to maintain efficacy in terms of prevention of systemic embolisms with a substantial cut down of bleeding complications by 40%.

Stent thrombosis rate has attracted much interest for several reason. In fact, while its low rate renders all single RCT underpowered, the clinical impact of a single event raises concerns among cardiologists. A recent meta-analysis reported a slightly higher, yet statistically borderline significant, Stent Thrombosis rate with a DAT regimen [13], which was mainly driven by results from the AUGUSTUS. However, they included all 4 treatment groups in the calculation, resulting in a comparison of dual versus triple treatment regardless if VKA or DOAC were used. This represents a substantial difference with all remaining trials, as they all compared a DAT regimen including a DOAC versus a TAT regimen including a VKA (representing the previous clinical standard). On the contrary, we maintained the same between-the-groups comparison across all included studies and could not find any significant difference in Stent Thrombosis rate among. Nevertheless, it should be remembered that the risk for Stent Thrombosis has a large interindividual variability, being influenced both by patient-related factors such as diabetes, chronic kidney disease and prior stent thrombosis, and by lesion-specific factors, such as location, number and length of treated lesions, lesion anatomy and procedural factors, like stent under-expansion, geographic miss and iatrogenic dissection [14]. Stent thrombosis rate was highest among patients receiving DAPT for only 1 month in the PIONEER AF-PCI. Of note, in the same study stent thrombosis rate was more than doubled in the DAT versus the TAT arm both for patients receiving DAPT for 1 month and in those receiving DAPT for as long as 6 months but not in those on DAPT for 12 months. Of note, in the AUGUSTUS Trial both TAT regimens either with DOAC or VKA were associated with a lower Stent Thrombosis rate than the respective DAT regimens [5]. This result needs to be interpreted with caution and Stent Thrombosis risk should be assessed individually and weighted against the bleeding risk whose prognostic impact is similar when not larger than ischemic events [15–17].

The varying results observed regarding bleeding events among the studies included merits some considerations. First, it partially reflects the differences among the populations included in the four trials, such as the prevalence of hypertension and diabetes, the baseline HAS-BLEED score and the proportion of ACS patients. In addition, differences in study protocols have added further variability. For example, the time in therapeutic range was variable. Accordingly, we found a significant interaction between the primary safety endpoint and the proportion of patients with an INR below 2 at metaregression analysis. Furthermore, the time from the index coronary procedure and randomization was different between the studies and we found an interaction with the primary efficacy endpoint. In line with this observation, the ENTRUST AF-PCI trial, the one with the shortest time between index procedure and randomization was the only one not reaching the superiority threshold of this endpoint [6]. This suggests caution in the management of antithrombotic treatment during the first days after the index procedure, especially in ACS patients. Particular attention should be applied during overlapping phases between different anticoagulants.

A relationship has traditionally been reported between ischemic and bleeding risks, suggesting that those patients who might benefit the more from longer or more intensive antithrombotic treatment are unfortunately also exposed to a higher bleeding risk [18]. On the contrary, it has recently emerged that early discontinuation of DAPT might lead to a significant reduction of bleeding, without a relevant impact on ischemic risk [19]. The plot thickens with AF patients with indication for long-term anticoagulation. In this context, results from RCTs and the present meta-analysis suggest that DAT might be preferable to TAT to contain bleeding. However, in situations where concerns about the ischemic risk prevail, such as ACS, the option to adopt TAT might be considered, even though this decision should be carefully weighed against the potential increase in bleeding risk. In fact, we found a significant interaction between the number of ACS patients enrolled in the studies included and the effect size of the primary safety endpoint, suggesting that the benefit observed with DAT in terms of bleeding reduction over TAT is larger for ACS patients compared to stable CAD patients undergoing PCI. On the other hand, no similar interaction was observed for the primary efficacy endpoint, a composite of ischemic events. Altogether, these results suggest that a less intensive antithrombotic regimen and the use of DOACs are able to significantly reduce bleeding events and indeed to a larger extent in ACS patients, apparently without a payoff in terms of ischemic events.

This metanalysis has some limitations. The lack of double-blinding might represents a limitation. Notwithstanding the practical challenges and ethical concerns related to the use of a sham intervention, it remains unclear to what extent this determined a bias in the original studies and the present analysis. Clopidogrel was the P2Y_12_ inhibitor used in the majority of cases across all studies, thus these results may not apply to the currently used more effective P2Y_12_ inhibitors, which would have an impact on both safety and efficacy [20–21]. Finally, since all DAT patients underwent a variable peri-procedural period of triple therapy, the unavailability of patient-level data did not allow to account for this potentially relevant source of variability.

In conclusion, our results demonstrate the superiority of a DAT regimen including a DOAC to prevents bleeding events in patients with atrial fibrillation undergoing PCI, compared to a TAT regimen including a VKA with no increase in thrombo-embolic cardiovascular events.

## Supporting information

S1 Table Risk of bias of individual studies by Cochrane risk assessment tool. (DOCX)

S1 Checklist PRISMA checklist. (PDF)
