# Supplementary material for: Dual anti-thrombotic treatment with direct anticoagulants improves clinical outcomes in patients with Atrial Fibrillation with ACS or undergoing PCI. A systematic review and meta-analysis
Source: PLoS One. 2020 Jul 9;15(7):e0235511. doi: 10.1371/journal.pone.0235511 (PMC7347192; doi:10.1371/journal.pone.0235511)
Supplement: S1 Table — (DOCX) [file pone.0235511.s001.docx]

**Supplementary Table S1. Risk of bias of individual studies by Cochrane risk assessment tool**

|  | PIONEER AF-PCI | RE-DUAL PCI | AUGUSTUS | ENTRUST AF-PCI |
| --- | --- | --- | --- | --- |
| Random sequence generation  *(Selection bias)* | 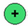 | 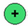 | 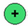 | 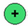 |
| Allocation concealment *(Selection bias*) | 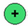 | 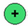 | 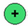 | 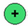 |
| Blinding of participants and personnel *(Performance bias)* * | 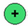 | 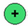 | 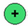 | 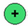 |
| Incomplete outcome data *(Attrition bias)* | 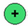 | 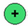 | 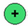 | 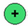 |
| Selective reporting *(Reporting bias)* | 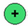 | 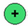 | 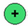 | 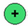 |
| Other sources of bias | 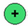 | 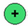 | 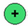 | 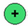 |

* All four trials were open label, however, since the outcome assessment was blinded, our judgement is that open label design has not influenced outcomes.
